# Non-Antimicrobial Drugs: Etodolac as a Possible Antimicrobial or Adjuvant Agent Against ESKAPE Pathogens

**DOI:** 10.2174/1874285801812010353

**Published:** 2018-10-23

**Authors:** Sónia G. Pereira, Vanessa S. Domingues, João Theriága, Maria de Jesus Chasqueira, Paulo Paixão

**Affiliations:** 1 CEDOC – Chronic Diseases Research Center, NOVA Medical School, NOVA University, Lisboa, Portugal; 2 Faculty of Pharmacy, University of Coimbra, Coimbra, Portugal

## Non-Antimicrobial Drugs: Etodolac as a Possible Antimicrobial or Adjuvant Agent Against ESKAPE Pathogens

The Open Microbiology Journal, **2018**, 12: 288-296

The correct numbering of author ‘s affiliation is mentioned below:

Sónia G. Pereira^1^, *, Vanessa S. Domingues^2^, João Theriága^1^, Maria de Jesus Chasqueira^1^, Paulo Paixão^1^

The original numbering of author ‘s affiliation provided was: 

Sónia G. Pereira^1^, *, Vanessa S. Domingues^2^, João Theriága^2^, Maria de Jesus Chasqueira^2^, Paulo Paixão^2^

